# Mindfulness and Basic Hope in Patients with Pelvic Cancer: Examining Illness Acceptance and Fear of Recurrence Within a Multiple Mediation Model

**DOI:** 10.3390/brainsci16050503

**Published:** 2026-05-05

**Authors:** Dariusz Krok, Ewa Telka, Sebastian Binyamin Skalski-Bednarz, Mariusz G. Kuźniar

**Affiliations:** 1 Department of Psychology, University of Opole, 45-052 Opole, Poland; 2 Department of Radiotherapy, Maria Sklodowska-Curie National Research Institute of Oncology, Gliwice Branch, 44-101 Gliwice, Poland; etelka@io.gliwice.pl; 3 Faculty of Philosophy and Education, Catholic University of Eichstätt-Ingolstadt, 85072 Eichstätt, Germany; sebastian.skalski@ku.de; 4 Institute of Psychology, Ignatianum University in Cracow, 31-501 Kraków, Poland; 5 Department of Psychosocial Sciences and Ethics, Charles University, Prague 1, 162 52 Prague, Czech Republic; mariusz.kuzniar@htf.cuni.cz

**Keywords:** mindfulness, illness acceptance, fear of recurrence, basic hope, pelvic cancer, multiple mediation

## Abstract

**Highlights:**

**What are the main findings?**

- Mindfulness was positively associated with basic hope among patients with pelvic cancer.
- Mindfulness was positively related to all dimensions of illness acceptance, and negatively related to fear of recurrence.
- Illness acceptance functioned as a significant mediator between mindfulness and basic hope.
- Fear of recurrence was not a mediator between mindfulness and basic hope.
- Illness acceptance and fear of recurrence were sequential mediators linking mindfulness to basic hope.

**What are the implications of the main findings?**

- Acceptance-related processes represent a central mechanism through which mindfulness is related to basic hope in cancer patients.
- Lower fear of recurrence may be indirectly linked to basic hope through higher illness acceptance.
- Integrating cognitive, emotional, and existential perspectives is crucial in meaningful and hopeful perceptions of life in the context of chronic illness.
- Mindfulness-based interventions may be effective in promoting adaptive psychological adjustment in oncology settings.

**Abstract:**

**Background/Objectives**: Mindfulness has been identified as a protective factor in promoting adaptive psychological outcomes among cancer patients, yet the mechanisms linking mindfulness to fundamental existential beliefs, such as basic hope, remain underexplored. In addition, mediational processes underlying these relationships remain understudied. Building on the theoretical framework of Acceptance and Commitment Therapy, we tested a serial multiple mediation model in which illness acceptance and fear of recurrence sequentially mediated the association between mindfulness and basic hope. **Methods**: Adult patients diagnosed with pelvic cancer (N = 273) who were undergoing oncological treatment completed questionnaires measuring mindfulness, illness acceptance, fear of recurrence, and basic hope. Mediation analysis was used to examine multiple mediation effects. **Results**: Illness acceptance also functioned as a single mediator between mindfulness and basic hope. In contrast, fear of recurrence was not a mediator between mindfulness and basic hope. The main finding was the serial mediation pathway through illness acceptance and fear of recurrence. The indirect effects showed that mindfulness was positively associated with illness acceptance dimensions—life satisfaction during illness, reconciliation with the disease, and self-distancing from the disease—which, in turn, were negatively associated with fear of recurrence, ultimately relating to higher levels of basic hope. **Conclusions**: The findings highlight the critical role of acceptance- and fear-related processes in sustaining basic hope among cancer patients and suggest that mindfulness-based interventions may foster adaptive adjustment to illness. Implications for clinical practice include integrating mindfulness and acceptance-focused strategies to enhance hope and support emotional well-being in patients coping with cancer.

## 1. Introduction

### 1.1. Mindfulness and Basic Hope in Struggling with Cancer

Cancer-related cognitions and emotions constitute a major challenge in patients’ lives, affecting not only mental health and quality of life but also fundamental beliefs about the world and its organizational structure. The diagnosis of cancer and subsequent treatment represent life-changing events that influence a wide array of psychological processes and shape future life trajectories [1,2]. Unsurprisingly, the way in which patients experience and process their current situation—both cognitively and emotionally—significantly moderates their global perception of themselves and the surrounding world. Mindfulness constitutes an important psychological resource in the adjustment to pelvic cancer, a group of conditions often associated with significant physical burden and psychosocial challenges [3]. Mindfulness, typically defined as nonjudgmental awareness of present-moment experiences, has been linked to lower psychological distress, including lower levels of pain catastrophizing [3] and anxiety [4], and higher emotional well-being and social support [5]. It may also facilitate adaptive coping by enhancing emotional regulation, decreasing rumination, and promoting acceptance of illness-related experiences. Empirical evidence suggests that dispositional mindfulness—that is, an individual’s tendency to maintain present-moment awareness with an attitude of openness, acceptance, and non-judgment—enables cancer patients to cope with the negative consequences of illness [6], regulate distressing thoughts and emotions [7], and improve overall psychological well-being [8].

Hope—especially in its basic or existential form that reflects a fundamental worldview, formed early in life, that the world is generally meaningful, ordered, and positive [9,10,11]—plays a significant role in patients with pelvic cancer as it may buffer against despair and helplessness, support engagement in treatment and foster a sense of future orientation even under conditions of uncertainty. Research showed that higher levels of hope were associated with lower procedural distress [12], better psychological adjustment and improved quality of life [13]. Assessing mindfulness and hope in pelvic cancer patients is therefore important, as these factors are likely to serve as protective factors that mitigate distress and promote psychological well-being. Their measurement allows for the identification of individuals at risk of poorer adjustment and may inform the development of targeted psychosocial interventions aimed at enhancing adaptive coping and overall quality of life.

Correlational and longitudinal studies have shown that dispositional mindfulness positively contributes to hope among patients with cancer. For example, mindfulness was positively associated with higher levels of hope in women experiencing cancer recurrence [14] as well as in breast cancer patients and survivors [15]. Longitudinal research has further demonstrated the beneficial effects of mindfulness-based interventions on hope. Mindfulness strategies turned out to increase hope, conceptualized within Snyder’s framework, among cancer patients undergoing radiotherapy [16]. Mindfulness-based stress reduction techniques improved hope in breast cancer patients, suggesting that mindfulness meditation and mind–body awareness enhance goal-directed motivation and pathways [17]. Additionally, a four-week Mindfulness-Integrated Cognitive Behavioral Therapy intervention increased hope in women with stage I–III breast cancer undergoing chemotherapy, fostering a more positive attitude during the mentally taxing treatment process [18]. Collectively, these findings indicate that mindfulness consistently contributes to daily experiences of hope in cancer patients and survivors, both during treatment and in the survivorship phase.

Although these studies provide strong evidence for a relationship between mindfulness and hope, none examined basic hope as a distinct construct. Importantly, hope conceptualized by Snyder [19] and basic hope, based on Erikson’s approach [10], are related but conceptually distinct. Their differences lie primarily in theoretical foundations, psychological functions, and levels of generality [11]. Snyder’s hope is a cognitive-motivational construct related to goal pursuit, encompassing agency and pathways, whereas basic hope is generally viewed as a fundamental, emotional, and existential belief about the world as meaningful, ordered, and positive. This structural distinction is particularly relevant in the context of examining psychological resources among cancer patients.

Research on basic hope among cancer patients remains limited. One study investigated the relationships among cancer recurrence, pain-control self-efficacy, pain-coping strategies, and basic hope across different cancer types. Results indicated that higher self-efficacy was positively associated with basic hope, with men exhibiting greater self-efficacy than women. Moreover, effective pain control and the use of pain-coping strategies were positively related to basic hope [20]. However, another study of women with breast cancer found that basic hope was not significantly associated with disease adaptation [21]. This inconsistency suggests that basic hope may not directly translate into adaptive functioning in all clinical populations and may instead operate through mediational factors.

Basic hope has also been investigated in non-cancer populations. For instance, post-traumatic growth was positively associated with basic hope in individuals who experienced severe physical injuries or other traumatic events (e.g., assault, rape) [22]. Research on women with physical disabilities further revealed significant negative associations between basic hope and certain coping strategies, including problem-focused coping and alcohol use. Moreover, among women with physical disabilities, the relationship between basic hope and coping was moderated by the type of disability: higher basic hope was associated with greater use of problem-focused coping in those with acquired disabilities, and with lower use of substance-related coping in those with congenital disabilities [23]. In summary, these findings highlight gaps in research on basic hope among cancer patients and underscore the need to investigate potential factors, such as mindfulness, that may contribute to or mediate the development of basic hope in this population.

### 1.2. Illness Acceptance and Fear of Recurrence as Psychologically Functional Factors in Cancer

Previous research suggests that the relationship between mindfulness and hope is not direct but may be influenced by intermediary psychological processes, such as illness acceptance and fear of recurrence. This assumption is supported by both the theoretical framework of Acceptance and Commitment Therapy (ACT) and empirical findings. Within the ACT framework, developed by Hayes and colleagues [24,25], the relationship between mindfulness and hope can be conceptualized in terms of psychological flexibility, defined as the ability to remain in contact with the present moment with openness and awareness while engaging in value-consistent behavior despite the presence of distress (e.g., serious illness). Mindfulness is associated with higher awareness and lower experiential avoidance, thereby facilitating the acceptance of illness-related uncertainty, intrusive thoughts, and negative emotions [26]. By promoting such acceptance, mindfulness is associated with overall adaptation to illness, lower threat-related appraisals and ruminative tendencies, and weaker negative emotional responses to stress [27]. Consequently, individuals are better able to maintain optimistic beliefs about their lives and the surrounding world in terms of meaningfulness and order [28]. From this perspective, mindfulness may support the development or maintenance of hope by fostering acceptance of difficult experiences and enabling disengagement from rigid cognitive and emotional patterns, thereby preserving an existential, belief-based sense of hope, particularly in the context of serious illness.

Empirical findings further support the mediational role of illness acceptance in oncology populations. Among Malaysian patients with various cancers, illness acceptance mediated the relationship between cancer-related complaints and psychological distress: more complaints were associated with poorer illness acceptance, which, in turn, predicted higher distress [29]. Similarly, in advanced lung cancer patients, illness acceptance mediated the associations between personality traits (neuroticism and extraversion) and depression: higher neuroticism was linked to lower illness acceptance, which was associated with higher depression, whereas higher extraversion predicted greater illness acceptance and lower depression [30]. In elderly Chinese patients with breast cancer, illness acceptance mediated the relationship between family care and self-perceived burden, with serial mediation including perceived control also confirmed [31]. Other studies demonstrated direct positive associations between illness acceptance and hope in patients with lung cancer [32] as well as across different cancer types [33].

Fear of recurrence is another important factor influencing hope, particularly among patients with pelvic cancer, with approximately 56% reporting high levels [34,35]. Fear of recurrence has been shown to mediate clinical outcomes in cancer populations. In patients with head and neck cancer, it mediated the relationship between symptom severity and quality of life: higher symptom severity was associated with greater fear of recurrence, which in turn predicted lower quality of life [36]. In women with breast cancer, fear of recurrence mediated the relationship between fatalistic beliefs and both depressive and anxiety symptoms [37]. More complex, serial mediation models have also been identified: for instance, fear of recurrence, together with meaning-making, serially mediated the relationship between social support and illness acceptance in breast cancer patients [38], and in patients with thyroid cancer, fear of recurrence and symptom experiences serially mediated the relationship between voice changes and voice-related quality of life [39].

Collectively, these findings indicate that mindfulness may influence hope indirectly through cognitive, emotional, and behavioral mechanisms. However, the studies cited did not directly assess basic hope or examine the serial mediational effects of illness acceptance and fear of recurrence in patients with pelvic cancer, highlighting the need for further research in this population.

### 1.3. The Present Study

**Figure 1:**
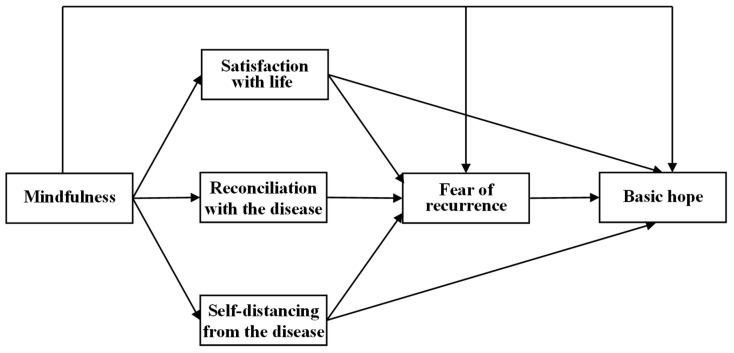
A theoretical model among the study variables.

The present study aimed to examine the relationships among mindfulness, illness acceptance, fear of recurrence, and basic hope in patients with pelvic cancer. Drawing on the ACT framework [24,25] and Mindfulness-Based Cancer Interventions [16,17], as well as previous research highlighting the protective role of mindfulness in fostering adaptive psychological outcomes in clinical populations [3,15,40], we investigated whether mindfulness would be associated with higher levels of basic hope and whether this relationship would be mediated by illness acceptance and fear of recurrence. Specifically, we proposed a serial multiple mediation model in which mindfulness was specified as the independent variable, basic hope as the dependent variable, and illness acceptance and fear of recurrence as sequential mediators. Additionally, we examined whether the individual dimensions of illness acceptance and fear of recurrence would function as parallel and serial mediators, providing a more detailed understanding of the mechanisms linking mindfulness to basic hope (Figure 1).

Based on prior theoretical and empirical evidence, the following hypotheses were formulated:

**Hypothesis** **1** **(H1):** *Higher levels of mindfulness will be directly associated with higher levels of basic hope.*

**Hypothesis** **2** **(H2):** *Illness acceptance will mediate the relationship between mindfulness and basic hope; specifically, higher mindfulness will be positively associated with basic hope indirectly through greater illness acceptance.*

**Hypothesis** **3** **(H3):** *Fear of recurrence will mediate the relationship between mindfulness and basic hope; specifically, higher mindfulness will be associated with lower fear of recurrence, which, in turn, will be associated with higher basic hope.*

**Hypothesis** **4** **(H4):** *Illness acceptance and fear of recurrence will act as sequential mediators in the relationship between mindfulness and basic hope; specifically, higher mindfulness will be positively associated with the dimensions of illness acceptance (parallel mediators), which, in turn, will be negatively associated with fear of recurrence (a serial mediator), ultimately relating to higher levels of basic hope.*

## 2. Materials and Methods

### 2.1. Participants

The study sample consisted of 273 adult patients (131 women and 142 men) diagnosed with pelvic cancers who were undergoing active oncological treatment (i.e., radiotherapy, chemotherapy, or combined therapy) [41]. From a medical perspective, pelvic cancers encompasses a range of distinct oncological conditions, including organs located within the pelvic cavity, most commonly including cancers of the prostate, bladder, rectum, cervix, endometrium, and ovaries [41]. These diagnoses differ meaningfully with respect to disease course, prognosis, treatment modalities, and psychosocial consequences, including issues related to sexual functioning, body image, and fear of recurrence. Although these cancers are etiologically diverse, they are often considered together in clinical and psycho-oncological research due to several shared characteristics in etiology (e.g., comparable patterns of local disease progression), prognosis (similar risks of local recurrence and distant metastasis), and common psychological challenges (e.g., concerns related to bodily integrity, identity, or sexual functioning).

### 2.2. Inclusion and Exclusion Criteria

Participants were eligible for inclusion if they met the following criteria: (1) a confirmed diagnosis of pelvic cancer at stages I–III; (2) sufficient cognitive capacity to understand the study procedures and independently complete the questionnaire measures; and (3) active participation in oncological treatment, specifically radiotherapy or chemotherapy. Exclusion criteria included: (1) receipt of palliative care or exclusively palliative treatment; (2) the presence of severe comorbid medical conditions that could compromise the reliability of self-reported responses (e.g., advanced cardiovascular disease, significant communication difficulties, or stage IV cancer); or (3) metastases located outside the pelvic region. These criteria were applied to ensure clinical comparability of the sample and to minimize potential confounding factors that could influence patients’ psychological responses during treatment. Given that the patients were currently undergoing different types of oncological treatment (chemotherapy, radiotherapy, or combination therapy), we did not distinguish separate groups for the analyses. The time since diagnosis ranged from 2 months to 9 years (M = 2.20, SD = 2.80).

### 2.3. Procedure

Participants were recruited from oncological clinics and outpatient centers located in southern Poland. The recruitment process was conducted in collaboration with oncologists working in the participating medical units. Initially, oncologists screened medical records to identify patients who were scheduled to begin radiotherapy or chemotherapy and who met the predefined eligibility criteria. During routine treatment consultations, eligible patients were informed about the opportunity to participate in the study by trained research personnel. Patients who expressed interest were provided with additional information and given the opportunity to ask questions before making their decision. Those who agreed to participate received detailed information regarding the study’s purpose, the voluntary nature of participation, and the research procedures. Written informed consent was obtained from all participants prior to data collection. Participation was entirely voluntary, and patients were informed that they could withdraw from the study at any stage without any consequences for their medical treatment.

**Figure 2:**
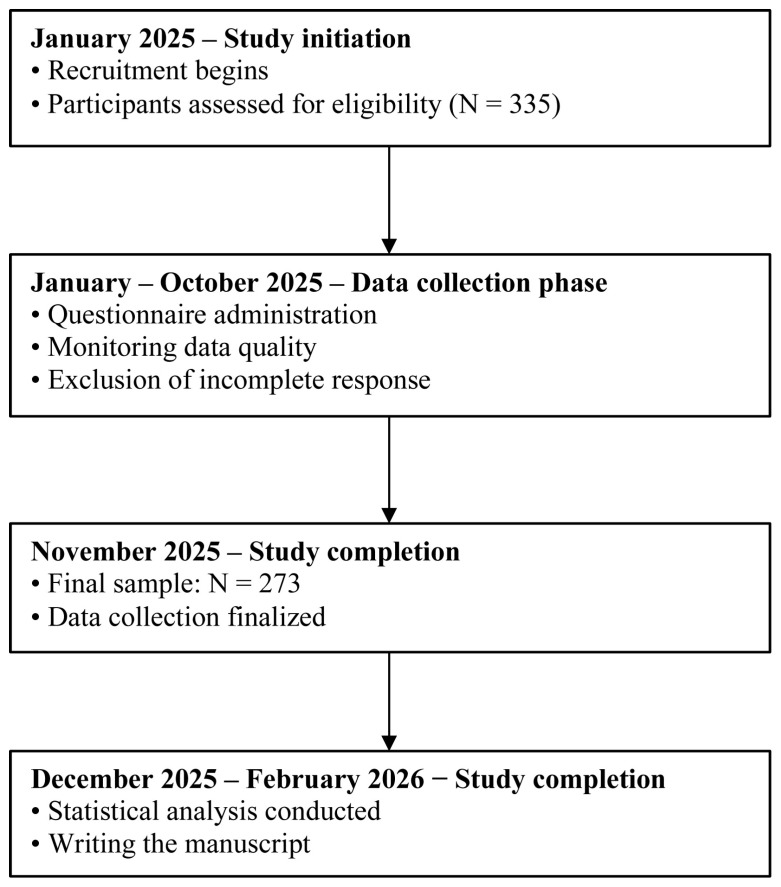
Study timeline.

In total, 335 patients were approached and invited to participate in the study. Of these, 29 individuals declined participation. An additional 31 patients were excluded due to medical conditions that emerged after the initial screening and rendered them ineligible according to the study criteria. Ultimately, 273 patients agreed to participate and completed the study measures, resulting in a final participation rate of 82.08% (Figure 2). The study was conducted according to the guidelines of the Declaration of Helsinki, and approved by The University Research Ethics Committee at the University of Opole (Protocol Code: UREC 64/2/2024; date of approval: 19 December 2024).

### 2.4. Measures

Mindfulness. To assess mindfulness we used the Freiburg Mindfulness Inventory (FMI), developed by Walach and colleagues [42] to measure dispositional mindfulness, defined as an individual’s tendency to maintain present-moment awareness and to approach internal experiences with an attitude of openness, acceptance, and non-judgment. The inventory includes 14 items describing everyday experiences related to mindful awareness and acceptance (e.g., attention to present experiences, openness to thoughts and emotions, and non-reactivity to internal events). Participants are asked to indicate the extent to which each statement applies to them using a four-point Likert scale, ranging from 1 (rarely) to 4 (almost always). The total score is calculated by summing responses across all items, with higher scores indicating greater levels of dispositional mindfulness. In the present study, the internal consistency was Cronbach’s α = 0.84, indicating satisfactory reliability.

Illness acceptance. The Acceptance of Life with the Disease Scale (ALDS) developed by Janowski and colleagues [43] was applied to assess the degree to which individuals psychologically accept their illness and integrate it into their everyday life. The scale emphasizes patients’ ability to adapt to their condition and maintain a meaningful and satisfactory life despite the presence of disease. It consists of 20 items, forming three subscales: (a) satisfaction with life, (b) reconciliation with the disease, and (c) self-distancing from the disease. The total score can be computed by summing responses across all items, with higher scores reflecting greater acceptance of life with the disease and better psychological adaptation to the illness. Participants rate each item using a four-point Likert-type response scale, indicating the extent to which each statement applies to their current experience, from 1 (no) to 4 (yes). In the present study, the internal consistency assessed by using Cronbach’s α ranged from 0.86 to 0.91.

Fear of Recurrence. To measure fear of recurrence the Cancer Worry Scale developed by Custers and colleagues [44]. The scale is a self-report instrument designed to measure the extent to which individuals experience worry or concern about the possibility of cancer returning or progressing. It consists of 8 items assessing different aspects of cancer-related fear of recurrence, including the frequency of thoughts about recurrence, the emotional impact of these thoughts, and the extent to which such worries interfere with daily functioning. Participants rate items on a four-point Likert scale, ranging from 1 (never) to 4 (almost always). The total score is calculated by summing responses to all items, with higher scores indicating a greater level of fear of recurrence. In the present study, the internal consistency was Cronbach’s α = 0.88.

Basic hope. The Basic Hope Inventory (BHI-12) developed by Trzebiński and Zięba [45] was used to assess a strength of basic hope. The inventory includes 12 items describing beliefs about the structure and meaningfulness of the world and the perceived likelihood of positive outcomes in life. Participants respond to each statement on a five-point Likert scale, ranging from 1 (strongly disagree) to 5 (strongly agree). The total score is calculated by summing responses across all items, with higher scores indicating higher levels of basic hope. In the present study, the internal consistency was Cronbach’s α = 0.76.

### 2.5. Data Analysis

Prior to conducting the main analyses, an a priori power analysis was performed using G*Power software 3.1. to determine the minimum sample size required to detect statistically meaningful effects in the mediation model [46]. The analysis indicated that a sample of at least 263 participants would be sufficient to detect a small effect size (f^2^ = 0.05) with a statistical power of 0.80 (1 − β), assuming five predictors and a significance level of α = 0.05. To enhance the statistical reliability of the results and decrease the potential risk of estimation errors, a slightly larger sample (N = 273) was included in the final analyses, thereby increasing the stability and precision of parameter estimates as well as the robustness of the mediation effects. As all variables were assessed using self-report questionnaires, Harman’s one-factor test was conducted to examine the potential presence of common method variance, which could otherwise bias the results [47] (Podsakoff et al., 2003). The analysis indicated that the items loaded onto 15 distinct factors, with the first unrotated factor accounting for only 22.19% of the total variance. These results suggest that common method variance is unlikely to be a significant concern in the present study. At the same time, we acknowledge that Harman’s single-factor test has important limitations and should not be treated as definitive evidence. Although the results suggest that common method variance is unlikely to be a major concern, its potential presence cannot be fully ruled out based on this test alone.

All statistical analyses were conducted using IBM SPSS Statistics (Version 28). First, descriptive statistics (means and standard deviations) were calculated for all study variables. Next, Pearson’s two-tailed correlation coefficients were computed to examine the preliminary relationships among mindfulness, illness acceptance, fear of recurrence, and hope. To test the hypothesized relationships between the study variables, a serial multiple mediation analysis was conducted. In this model, mindfulness was specified as the independent variable, hope as the dependent variable, and illness acceptance and fear of recurrence as sequential mediators.

The mediation analysis was performed using the PROCESS macro for SPSS [48]. Since we aimed to examine illness acceptance as a mediator at two levels—(1) as a global score and (2) as three specific components functioning as parallel mediators—together with an additional mediator, fear of recurrence, two models were employed. The first model (Model 6) enabled the examination of the overall illness acceptance score and fear of recurrence as serial mediators in the relationship between mindfulness and hope. The second model (Model 80) tested whether the three individual dimensions of illness acceptance, together with fear of recurrence, functioned as both parallel and serial mediators within the same model. This approach is complementary and allows for the testing of both serial mediation (i.e., involving two mediators) and more complex mediation structures, incorporating both parallel and serial indirect effects. The significance of both direct and indirect effects was evaluated using a bootstrapping procedure with 10 000 resamples and bias-corrected 95% confidence intervals (CIs).

Missing data were handled using case-wise mean substitution, allowing for the retention of cases while minimizing potential statistical bias. Although mean substitution is not the most advanced method for dealing with missing values, the proportion of missing data in the present study was very low and affected only a limited number of variables (i.e., only 11 digits/numbers; the percentage was less than 0.01%). Sensitivity analyses were conducted with complete cases only. Given this minimal level of missingness, we considered that more complex imputation procedures would not substantially alter the results.

## 3. Results

### 3.1. Descriptive and Preliminary Statistics

The overall means, standard deviations, and correlations were computed to examine relationships among the study variables. They are presented in Table 1.

Most relationships among the study variables were statistically significant. Age was negatively associated with life satisfaction during illness and fear of recurrence, while it was positively associated with hope. Illness duration was positively related to fear of recurrence and negatively related to hope. Mindfulness showed positive associations with all dimensions of illness acceptance—life satisfaction during illness, reconciliation with the illness, self-distancing from the illness, and the overall illness acceptance score—as well as with hope, whereas it was negatively associated with fear of recurrence. In turn, all dimensions of illness acceptance and the overall illness acceptance score were negatively associated with fear of recurrence and positively associated with hope. Finally, fear of recurrence was negatively associated with hope.

### 3.2. Multiple Mediation Analyses

First, serial mediation analysis with 10,000 iterations of bootstrapping and 95% bias-corrected confidence intervals [48] investigated whether the overall illness acceptance score and fear of recurrence serially mediated the relationship of mindfulness with hope (Model 6). The results of direct, indirect and total effects are shown in Table 2.

Regarding direct effects, mindfulness was positively associated with illness acceptance but not directly associated with fear of recurrence, suggesting that higher levels of mindfulness are primarily linked to greater illness acceptance. Illness acceptance was negatively associated with fear of recurrence, indicating that higher acceptance of the illness corresponds to lower levels of fear of recurrence. Additionally, both mindfulness and illness acceptance positively predicted hope, whereas fear of recurrence negatively predicted hope. These findings suggest that higher levels of mindfulness and illness acceptance, together with lower levels of fear of recurrence, are associated with greater hope.

With respect to indirect effects, the results indicated that mindfulness was related to hope through multiple pathways. The first mediation effect showed that illness acceptance functioned as a single mediator in the relationship between mindfulness and hope: mindfulness was positively associated with illness acceptance, which, in turn, was positively associated with hope. Although there were direct associations among mindfulness, fear of recurrence, and basic hope, the second mediation model with fear of recurrence as a mediator turned out to be statistically nonsignificant. In the third mediation model, illness acceptance and fear of recurrence operated as sequential mediators. Specifically, higher mindfulness was associated with greater illness acceptance, which was related to lower fear of recurrence, which, in turn, was associated with higher levels of hope. The total indirect effect was also significant, suggesting that both mediators jointly contributed to the relationship between mindfulness and hope. Finally, the total effect of mindfulness on hope was significant, indicating an overall positive association that was partially explained by the indirect effects through illness acceptance and the sequential pathway involving fear of recurrence.

To examine differences among the mediated effects, the effect-contrast method was applied. The contrast between the simple mediation effects through illness acceptance and fear of recurrence was not statistically significant (Effect = 0.05, 95% CI [−0.02, 0.12]), indicating that neither mediator was significantly stronger than the other when considered independently. Similarly, the difference between the simple mediation through illness acceptance and the serial mediation via illness acceptance and fear of recurrence was not significant (Effect = −0.02, 95% CI [−0.10, 0.06]), suggesting that including fear of recurrence in the pathway did not substantially alter the indirect effect. In contrast, the difference between the simple mediation through fear of recurrence and the serial mediation via illness acceptance and fear of recurrence was significant (Effect = −0.07, 95% CI [−0.14, −0.02]). This indicates that fear of recurrence contributes more strongly to the relationship between mindfulness and hope when it operates in conjunction with illness acceptance rather than as an independent mediator.

In addition, a combined parallel and serial mediation analysis with 10,000 bootstrap resamples and 95% bias-corrected confidence intervals [48] was conducted to examine whether the individual dimensions of illness acceptance and fear of recurrence mediated the relationship between mindfulness and hope, while simultaneously testing the three parallel and sequential pathways. Model 80 was specified, in which the dimensions of illness acceptance functioned as parallel mediators and fear of recurrence served as a serial mediator. The results are presented in Table 3.

The results of the direct effects indicated that mindfulness was positively associated with all three dimensions of illness acceptance: life satisfaction during illness, reconciliation with the illness, and self-distancing from the illness. These findings suggest that higher levels of present-moment awareness are related to greater acceptance of the illness across its various psychological dimensions. In contrast, mindfulness was not significantly associated with fear of recurrence. The illness acceptance dimensions were negatively related to fear of recurrence, indicating that greater acceptance, reconciliation, and self-distancing in the context of illness were associated with lower levels of fear of recurrence. With regard to predictors of hope, mindfulness demonstrated a positive direct effect on hope, indicating that higher levels of present-moment awareness were associated with higher levels of hope. However, the dimensions of illness acceptance were not significantly related to hope directly. In contrast, fear of recurrence was negatively associated with hope, suggesting that higher levels of fear regarding illness recurrence are linked to lower levels of hope.

Furthermore, significant serial mediation effects were observed, indicating that mindfulness was related to hope through illness acceptance and subsequently through fear of recurrence. Specifically, the three dimensions of illness acceptance—life satisfaction during illness, reconciliation with the illness, and self-distancing from the illness—and fear of recurrence sequentially mediated the relationship between mindfulness and hope. A more detailed analysis revealed that mindfulness was positively associated with each dimension of illness acceptance, which, in turn, were negatively associated with fear of recurrence; lower levels of fear of recurrence were subsequently associated with higher levels of basic hope. Among these pathways, the strongest mediation effect was observed for self-distancing from the illness. The total indirect effect was also significant, indicating that the mediators collectively accounted for a meaningful portion of the relationship between mindfulness and hope.

The total effect of mindfulness on hope was significant, suggesting that mindfulness contributes to higher levels of hope both directly and indirectly through the identified mediating mechanisms. Finally, to examine differences between specific indirect effects, the effect-contrast method was applied. However, none of the contrasts reached statistical significance (as the confidence intervals included zero), indicating that the mediation effects were of comparable magnitude. We also checked our mediation models for potentially relevant covariates (i.e., sex, age, and illness duration). However, the results remained largely consistent with the original findings, indicating that the observed associations are robust even after controlling for these demographic and clinical factors (all *p* values > 0.05).

**Figure 3:**
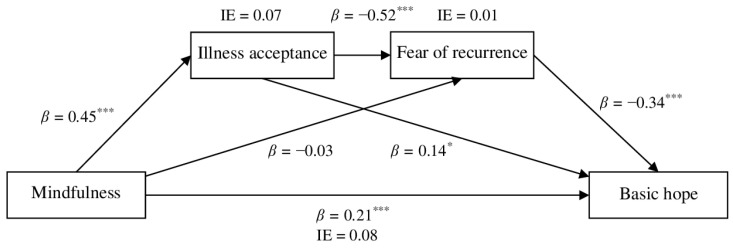
The final mediation model of illness acceptance and fear of recurrence in associations between mindfulness and basic hope (standardized coefficients β, and indirect effects IE); * *p* < 0.05, *** *p* < 0.001.

**Figure 4:**
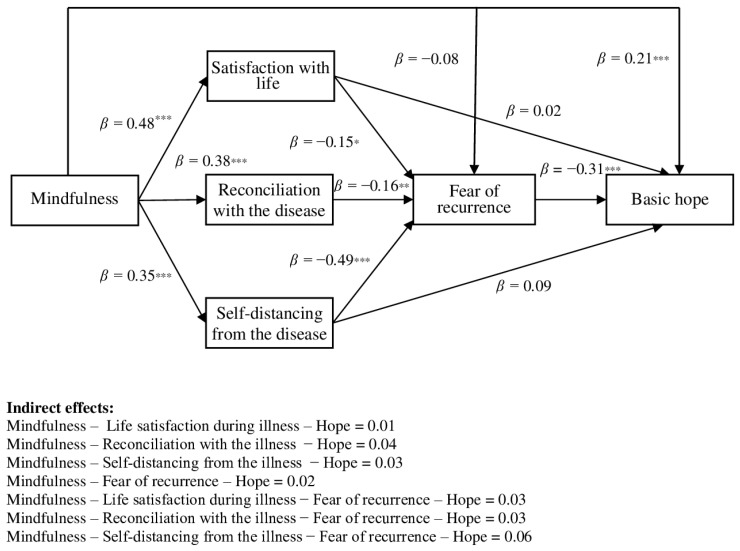
The final mediation model of illness acceptance dimensions and fear of recurrence in associations between mindfulness and basic hope (standardized coefficients β, and indirect effects IE); * *p* < 0.05, ** *p* < 0.01, *** *p* < 0.001.

The graphic representation of the direct and indirect results are shown in Figure 3 and Figure 4.

## 4. Discussion

The present study investigated the relationships between mindfulness and basic hope within a multiple mediation model involving illness acceptance and fear of recurrence among patients with pelvic cancer. The results of the mediation analyses largely supported the proposed hypotheses. To the best of our knowledge, this is the first study to jointly examine both parallel and serial mediational pathways linking cancer patients’ tendency to maintain present-moment awareness with their fundamental beliefs about the world’s meaningfulness, order, and positive existential qualities in a sample of patients with pelvic cancer. As such, these findings extend the ACT framework and provide novel insights into the psychological mechanisms underlying the effects of mindfulness on basic hope.

### 4.1. Direct Associations Between Mindfulness and Basic Hope

As hypothesized, we found robust evidence for a positive association between mindfulness and basic hope among patients with pelvic cancer (H1). Patients characterized by non-judgmental, present-moment awareness were more likely to perceive the world as meaningful, ordered, and generally benevolent. These findings are consistent with previous research indicating that a greater tendency to maintain present-moment awareness, combined with an attitude of openness, acceptance, and non-judgment, is associated with higher confidence in one’s ability to achieve desired outcomes among women with cancer recurrence [14], breast cancer patients [15], and individuals undergoing radiotherapy for various types of cancer [16].

However, the present study extends prior research by demonstrating a positive association between mindfulness and basic hope, a construct that differs conceptually from the form of hope proposed by Snyder [19] and employed in earlier studies [14,15]. This finding highlights the distinctive role of basic hope as a fundamental cognitive–existential resource in the context of chronic illness. Unlike more situational and goal-oriented forms of hope, basic hope reflects a generalized belief in the meaningfulness, order, and coherence of the world, which may be particularly challenged by the experience of cancer [21]. The observed positive association between mindfulness and basic hope may suggest that mindfulness may contribute to maintaining or reconstructing these core assumptions, even in the face of illness-related uncertainty and distress. Specifically, among cancer patients experiencing elevated levels of uncertainty and psychological distress, the ability to deliberately direct attention to the present moment—without judgment or excessive cognitive elaboration—may facilitate the development of hope-related beliefs concerning the meaningfulness and comprehensibility of the world.

Given that disruptions in belief systems, meaning structures, and life goals are common among cancer patients and are often associated with increased psychological distress [2,49], and higher basic hope through mindfulness-related processes may play a crucial role in supporting emotional stability and adaptive coping with serious health conditions. This interpretation is supported by previous findings demonstrating the importance of basic hope in the psychological functioning of cancer patients, particularly in relation to the use of personal resources and coping strategies [20]. Mindfulness may thus help patients with pelvic cancer maintain a coherent and meaningful perspective on the world and their future, even in the context of illness uncertainty and distress.

### 4.2. Single Mediational Effects of Illness Acceptance and Fear of Recurrence

As hypothesized, illness acceptance mediated the relationship between mindfulness and basic hope, such that higher levels of mindfulness were associated with greater illness acceptance, which, in turn, was related to stronger basic hope (H2). For patients with pelvic cancer, this finding may suggest that mindfulness is associated with basic hope indirectly through its positive association with illness acceptance. At the same time, this result is consistent with previous studies demonstrating the mediating role of illness acceptance in different contexts, such as the relationship between cancer-related symptoms and psychological distress in patients with various types of cancer [29], as well as the relationship between personality traits and depression in patients with advanced lung cancer [30].

However, our findings also contribute to a broader understanding of the mediating role of illness acceptance in the psychological functioning of cancer patients. Specifically, they indicate that an accepting and mature approach to chronic illness may mediate not only the relationships between physiological cancer symptoms or personality traits and psychological outcomes, but also the relationship between the capacity for mindful, present-moment awareness and a more global perception of experiences as meaningful and coherent. This underscores the importance of supporting cancer patients in developing a constructive and realistic understanding of their illness, enabling them to accept the demanding and distressing consequences associated with their condition [31,32]. In line with the assumptions of the ACT framework [25], patients who are able to acknowledge and accept the reality of their illness—without excessive avoidance or emotional reactivity—are more likely to utilize their capacity for open, present-moment awareness to maintain an optimistic and hopeful perspective on both their lives and the surrounding world [27,28]. This mediation pattern may suggest that mindfulness is not directly associated with basic hope but rather exerts its effect by promoting more adaptive cognitive–emotional adjustment to illness. This conclusion aligns with results obtained by Wang et al. [50], which highlighted the significant role of mindfulness in illness acceptance through resilience-related mechanisms. Mindfulness is likely to foster adaptive coping by enhancing individuals’ capacity to tolerate distress, maintain emotional balance, and reframe illness-related experiences in a more constructive manner.

Our results did not support the third hypothesis (H3), which posited a simple mediating effect of fear of recurrence in the relationship between mindfulness and basic hope. Despite significant correlations among mindfulness, fear of recurrence, and basic hope, the indirect effect was nonsignificant. Therefore, fear of recurrence was not a mediator between mindfulness and basic hope. This finding stands in contrast with earlier studies by Tsay et al. [36], in which fear of recurrence mediated the relationship between symptom severity and quality of life among patients with head and neck cancer, and by Tsai et al. [37], who demonstrated a mediating role of fear of recurrence in the relationship between fatalistic beliefs and depressive and anxiety symptoms in women with breast cancer.

One possible explanation for this discrepancy lies in conceptual differences between the constructs examined in the present study and those investigated in prior research. As a present-moment–oriented process, mindfulness operates primarily through acceptance rather than through the direct reduction in fear. Within the ACT framework [25,26], mindfulness is associated with lower experiential avoidance and cognitive fusion, thereby promoting acceptance rather than directly targeting specific fears. Accordingly, mindfulness may be more strongly associated with hope through the enhancement of present-moment awareness and acceptance, regardless of the extent to which patients experience concerns about the future. This interpretation is supported by studies indicating that mindfulness is more consistently associated with greater illness acceptance [27] and higher psychological flexibility [51] in patients with chronic illness, rather than with substantial reductions in fear of recurrence per se [52]. Taken together, these findings may suggest that mindfulness is related to basic hope primarily through acceptance-based processes rather than through changes in fear of recurrence.

### 4.3. Multiple Mediational Effects of Illness Acceptance and Fear of Recurrence

The primary finding of the present study concerns the multiple mediating roles of illness acceptance and fear of recurrence in the relationship between mindfulness and basic hope. As hypothesized (H4), illness acceptance and fear of recurrence functioned as sequential mediators in this relationship. More specifically, mindfulness was positively associated with the dimensions of illness acceptance (parallel mediators: satisfaction with life, reconciliation with the disease, and self-distancing from the disease), which, in turn, were negatively associated with fear of recurrence (a serial mediator), ultimately relating to higher levels of basic hope. This joint mediating effect further supports the above-formulated interpretation regarding the absence of a simple mediating effect of fear of recurrence. The multiple mediational effects are particularly noteworthy, as they underscore the importance of examining illness acceptance and fear of recurrence as interrelated processes operating sequentially [31,35].

Consequently, the present findings extend prior research on the relationship between mindfulness and goal-oriented hope [15,16,27] by clarifying the specific roles of illness acceptance and fear of recurrence in relation to basic hope among patients with pelvic cancer. Although these constructs are conceptually distinct, their joint examination within the relationship between mindfulness and basic hope provides a more comprehensive understanding than considering each factor in isolation. Moreover, the combined effects support the notion that fear of recurrence operates jointly not only with previously examined cognitive factors (e.g., meaning-making) [38] or experiential factors (e.g., illness symptoms) [39], but also with cognitive–emotional processes such as mindfulness in patients with pelvic cancer. Importantly, these findings suggest that the interplay between acceptance-related processes and fear-related responses may constitute a key mechanism underlying the psychological functioning of patients with cancer. Illness acceptance may facilitate a more balanced and less threat-focused appraisal of the disease, which in turn may allow patients to maintain or reconstruct a sense of fundamental trust in the world, as reflected in basic hope.

From a psychological perspective, the co-occurrence of illness acceptance and fear of recurrence appears theoretically justified within the ACT framework [25,26]. For patients with pelvic cancer, mindfulness—conceptualized as present-moment, non-judgmental awareness—may facilitate acceptance processes, reflected in higher levels of illness acceptance (i.e., satisfaction with life, reconciliation with the disease, and self-distancing). These processes are likely to be associated with lower experiential avoidance and cognitive fusion, thereby enabling patients to engage more adaptively with their illness [6,40]. In turn, greater illness acceptance is associated with lower fear of recurrence, understood as a future-oriented manifestation of threat-related cognition. Lower fear of future uncertainty may subsequently be linked to broader existential beliefs, including basic hope. Accordingly, rather than attempting to control or eliminate negative illness-related experiences, patients may learn to accept them while remaining oriented toward personally meaningful life directions [33]. In that way, they will be able to acquire a more coherent and hopeful perception of the self and the world, even in the face of illness-related uncertainty and adversity. This assumption is supported by previous findings that have highlighted the importance of the ACT in feelings of helplessness and the well-being of cancer patients. Encouraging patients to engage with personally meaningful values despite the presence of distress may facilitate a shift from passive resignation toward more active and purposeful adjustment [53]. Overall, the observed multiple mediation pattern is consistent with ACT assumptions, suggesting that mindfulness is associated with basic hope indirectly through both illness acceptance and fear of recurrence.

Given the cross-sectional nature of the data in our study, an alternative interpretation of the observed relationships should also be considered. While the proposed model assumes that mindfulness is associated with illness acceptance, which in turn is related to fear of recurrence and ultimately to basic hope, the direction of these associations may operate differently. For example, higher mindfulness may be associated with lower fear of recurrence, which in turn may relate to higher levels of illness acceptance, ultimately relating to greater basic hope. Such bidirectional dynamics are consistent with transactional models of stress and coping, in which cognitive, emotional, and meaning-related processes continuously influence one another over time [39,54].

### 4.4. Limitations and Future Implications

Although the present study revealed meaningful mediational relationships between mindfulness and basic hope in patients diagnosed with pelvic cancer, several limitations should be acknowledged. First, the sample was based on a non-probability convenience sampling method and included only patients with pelvic cancer, which may limit the generalizability of the findings to other cancer types, stages, or chronic illnesses. Psychological processes, particularly those related to illness acceptance and fear of recurrence, may vary across different patient populations [29,31]. Future research should examine similar models in more diverse clinical groups. Furthermore, expanding the range of variables to include factors such as cancer type, treatment modality, disease location, quality of medical care, family support, and geographic region would enhance the explanatory power of the findings. The category of patients with pelvic cancer may encompass a range of diagnostically and clinically diverse conditions, which is likely to affect psychological functioning and adjustment processes. Therefore, the potential heterogeneity within the pelvic cancer group should be considered when interpreting the results.

The cross-sectional design of the study also precludes causal inference. Although the multiple mediation model was supported and may tentatively suggest directional pathways, longitudinal or experimental studies are necessary to confirm these relationships. The tested mediation model should be interpreted as a theoretically informed statistical model, which does not allow for causal inference but rather identifies patterns of associations consistent with the proposed framework. Alternative explanations are also possible, including reverse or reciprocal relationships among the variables (e.g., basic hope−illness acceptance−fear of recurrence–mindfulness or mindfulness−fear of recurrence−illness acceptance–basic hope). Future research should also incorporate a broader range of clinical and psychosocial covariates to better isolate the unique contributions of the examined psychological constructs and provide a more comprehensive understanding of the mechanisms involved [30,32].

Another limitation of the present study concerns the approach used for handling missing data, which was addressed using mean substitution [48]. Although this method allowed for the preservation of the full sample size and ensured the completeness of the dataset, it is generally not an optimal strategy as it may reduce variance and potentially bias parameter estimates. However, due to a very small proportion of missing data in the current study, the procedure of mean substitution did not substantially alter the results.

Furthermore, the study focused on patients undergoing cancer treatment; thus, the findings may not generalize to individuals who have completed treatment, who may experience lower levels of fear of recurrence due to more favorable prognoses. Future research should therefore include cancer survivors to provide a broader perspective on these processes. Finally, the complexity of the relationship between fear of recurrence and basic hope requires further investigation. The absence of a significant simple mediating effect of fear of recurrence may suggest that its relationship with mindfulness and basic hope may be non-linear or moderated by other variables [22,23]. Future studies should incorporate additional factors (e.g., psychological distress or personality traits) to allow for a more nuanced understanding of these relationships.

Finally, all variables were assessed using self-report measures, which may be subject to common method bias, social desirability, and shared variance attributable to the measurement method. Although procedural and statistical steps were taken to minimize this risk, reliance on a single method of assessment may limit the robustness of the findings. The inclusion of multi-method approaches, such as clinician-rated measures or behavioral indicators, would strengthen the validity of future studies.

## 5. Conclusions

The present study provides novel insights into the psychological mechanisms linking mindfulness, illness acceptance, fear of recurrence, and basic hope among patients with pelvic cancer. Drawing on the ACT framework [25,26], the findings indicate that mindfulness is positively associated with basic hope, primarily through the multiple mediating effects of illness acceptance and fear of recurrence. This highlights the importance of integrating cognitive, emotional, and existential perspectives in fostering meaningful and hopeful perceptions of life in the context of chronic illness. From a clinical perspective, the findings support the integration of mindfulness- and acceptance-based interventions into psycho-oncological care [55]. Interventions aimed at enhancing mindfulness may be particularly effective when combined with strategies that promote illness acceptance and the regulation of anxiety and fear, as such approaches may help sustain fundamental hope-related beliefs in patients coping with cancer. Moreover, stress management programs that incorporate techniques for regulating anxiety and fear, as well as meaning-oriented and existential resources, may further contribute to improving patients’ psychological well-being in a clinical context. However, due to the cross-sectional nature of the data, the clinical implications should be regarded as tentative and hypothesis-generating rather than definite.

## Figures and Tables

**Table 1:** Correlations among age, illness duration, mindfulness, illness acceptance, fear of recurrence, and basic hope.

Variables	M	SD	1.	2.	3.	4.	5.	6.	7.	8.
Age	55.14	14.64	–							
2. Illness duration	2.20	2.80	0.04	–						
3. Mindfulness	2.90	0.54	0.07	0.02	–					
4. Life satisfaction during illness	3.26	0.61	−0.12 *	0.01	0.49 ***	–				
5. Reconciliation with the illness	3.15	0.66	−0.02	0.03	0.39 ***	0.79 ***	–			
6. Self-distancing from the illness	2.59	0.70	0.01	−0.03	0.35 ***	0.63 ***	0.72 ***	–		
7. Illness acceptance (Total score)	3.04	0.59	−0.09	0.01	0.45 ***	0.92 ***	0.90 ***	0.85 ***	–	
8. Fear of recurrence	2.70	0.70	−0.20 **	0.14 *	−0.27 ***	−0.40 ***	−0.53 ***	−0.60 ***	−0.54 ***	
9. Hope	3.62	0.84	0.22 ***	−0.17 **	0.37 ***	0.37 ***	0.42 ***	0.43 ***	0.43 ***	−0.48 ***

* *p* < 0.05; ** *p* < 0.01; *** *p* < 0.001; Abbreviations: M = Mean, SD = Standard Deviation.

**Table 2:** Mediation effects for the overall illness acceptance and fear of recurrence in the relationship of mindfulness with basic hope (standardized parameter estimates).

Direct Effects	Estimate	SE	*t*	Model *R*^2^
Mindfulness–Illness acceptance	0.45	0.06	8.30 ***	0.20 *** 0.20 ***
Mindfulness–Fear of recurrence	−0.03	0.07	−0.64	
Illness acceptance–Fear of recurrence	−0.52	0.07	−9.07 ***	0.29 *** 0.29 ***
Mindfulness–Hope	0.21	0.09	3.07 ***	
Illness acceptance–Hope	0.14	0.09	2.44 *	
Fear of recurrence–Hope	−0.34	0.07	−5.78 ***	0.31 *** 0.31 ***
Indirect effects	Effect	SE	LLCI	ULCI
Mindfulness–Illness acceptance–Hope	0.07	0.03	0.01	0.12
Mindfulness–Fear of recurrence–Hope	0.01	0.02	−0.03	0.05
Mindfulness–Illness acceptance–Fear of recurrence–Hope	0.08	0.02	0.05	0.12
Total indirect effect	0.16	0.03	0.08	0.23
Total effect				
Mindfulness–Hope	0.37	0.09	6.56 *	0.13 ***

* *p* < 0.05, *** *p* < 0.001; Abbreviations: SE = Standard Error, t = Student’s *t*-test, R^2^ = coefficient of determination, LLCI = Lower Level/Limit Confidence Interval, ULCI = Upper Level/Limit Confidence Interval.

**Table 3:** Mediation effects for the dimensions of illness acceptance and fear of recurrence in the relationship of mindfulness with basic hope (standardized parameter estimates).

Direct Effects	Estimate	SE	*t*	Model *R*^2^
Mindfulness–Life satisfaction during illness	0.48	0.06	9.13 ***	0.23 ***
Mindfulness–Reconciliation with the illness	0.38	0.07	6.93 ***	0.15 ***
Mindfulness–Self-distancing from the illness	0.35	0.06	6.06 ***	0.12 ***
Mindfulness–Fear of recurrence	−0.08	0.07	−1.36	
Life satisfaction during illness–Fear of recurrence	−0.15	0.10	−1.99 *	
Reconciliation with the illness–Fear of recurrence	−0.16	0.09	−2.96 **	
Self-distancing from the illness–Fear of recurrence	−0.49	0.07	−7.16 ***	0.39 ***
Mindfulness–Hope	0.21	0.09	3.62 ***	
Life satisfaction during illness–Hope	0.02	0.12	0.13	
Reconciliation with the illness–Hope	0.09	0.12	0.95	
Self-distancing from the illness–Hope	0.09	0.10	1.12	
Fear of recurrence–Hope	−0.31	0.08	−4.84 ***	0.31 ***
Indirect effects	Effect	SE	LLCI	ULCI
Mindfulness–Life satisfaction during illness–Hope	0.01	0.03	−0.10	0.11
Mindfulness–Reconciliation with the illness–Hope	0.04	0.04	−0.08	0.12
Mindfulness–Self-distancing from the illness–Hope	0.03	0.03	−0.03	0.09
Mindfulness–Fear of recurrence–Hope	0.02	0.02	−0.01	0.06
Mindfulness–Life satisfaction during illness–Fear of recurrence–Hope	0.03	0.01	0.01	0.05
Mindfulness–Reconciliation with the illness–Fear of recurrence–Hope	0.03	0.01	0.01	0.05
Mindfulness–Self-distancing from the illness–Fear of recurrence–Hope	0.06	0.02	0.03	0.09
Total indirect effect	0.15	0.04	0.08	0.23
Total effect				
Mindfulness–Hope	0.37	0.09	6.56 ***	0.13 ***

* *p <* 0.05, ** *p* < 0.01, *** *p* < 0.001; Abbreviations: SE = Standard Error, t = Student’s *t*-test, R^2^ = coefficient of determination, LLCI = Lower Level/Limit Confidence Interval, ULCI = Upper Level/Limit Confidence Interval.

## Data Availability

The data presented in this study are available at URL: https://osf.io/4mj5k/files/osfstorage (accessed on 26 March 2026).
